# Synthesis, Characterization, and Investigation of Corona Formation of Dipeptide-Based Nanomaterials

**DOI:** 10.3390/ma18010108

**Published:** 2024-12-30

**Authors:** Emrah Dikici, Burcu Önal Acet, Betül Bozdoğan, Ömür Acet, Inessa Halets-Bui, Dzmitry Shcharbin, Mehmet Odabaşı

**Affiliations:** 1 Scientific and Technological Application and Research Centre, Aksaray University, Aksaray 68100, Turkey; emrah.dikici25@gmail.com; 2 Faculty of Arts and Science, Chemistry Department, Aksaray University, Aksaray 68100, Turkey; brconl33@gmail.com (B.Ö.A.); betul.bozdogan@gmail.com (B.B.); 3 Vocational School of Health Science, Pharmacy Services Program, Tarsus University, Tarsus 33100, Turkey; omuracetbio@gmail.com; 4 Institute of Biophysics and Cell Engineering of the National Academy of Sciences of Belarus, 220072 Minsk, Belarus; inessahalets@gmail.com

**Keywords:** diphenylalanineamide dipeptide, nanomaterials, protein corona, self-assembly, biocompatibility

## Abstract

Peptide-based nanomaterials can be easily functionalized due to their functional groups, as well as being biocompatible, stable under physiological conditions, and nontoxic. Here, diphenylalanineamide-based nanomaterials (FFANMs) were synthesized, decorated with Ca^2+^ ions to set the surface charge, and characterized for possible use in gene delivery and drug release studies. FFANMs were characterized by SEM, TEM, dynamic light scattering (DLS), and LC-MS/MS. Corona formation and biocompatible studies were also carried out. Some of the data obtained are as follows: FFANMs have a diameter of approximately 87.93 nm. While the zeta potentials of FFANMs and Ca^2+^@FFANMs were −20.1 mV and +9.3 mV, respectively, after corona formation with HSA and IgG proteins, they were shifted to −7.6 mV and −3.7 mV, respectively. For gene delivery studies, zeta potentials of Ca^2+^@FFANMs and DNA interactions were also studied and found to shift to −9.7 mV. Cytotoxicity and biocompatibility studies of NMs were also studied on HeLa and HT29 cell lines, and decreases of about 5% and 10% in viability at the end of 24 h and 72 h incubation times were found. We think that the results obtained from this study will assist the groups working in the relevant field.

## 1. Introduction

Nanomaterials (NMs) are well-established integral components within various domains of science and technology. As the need for nanomaterials increases day by day, the global nanomaterials market size, estimated at approximately USD 9.4 billion in 2021, is expected to increase at a compound annual growth rate (CAGR) of 14.1% through 2028 [1]. Their unique characteristics, encompassing size, structure, and chemical properties, offer a wide spectrum of potential applications and innovative approaches for fundamental research. Particularly, NMs derived from diphenylalanine dipeptide and its derivatives have garnered growing interest due to their unique advantages in gene and drug delivery applications, notably in terms of biocompatibility, biodegradability, and surface modifiability [2,3]. The desired features of NMs designed for delivery applications include their appropriateness for circulating in the bloodstream and being internalized by cells. One of the most crucial physicochemical properties that influence their internalization into cells and intracellular trafficking is NM size. For delivery applications, the preferred NM size is approximately around 100 nm [4]. Diphenylalanineamide-based NMs have been previously fabricated in various sizes and have been employed as carriers for anticancer drugs (480 nm) [3], fluorescent dyes (280 nm) [5], photosensitive drugs (70–100 nm) [6], and fluorescent agents (84–163 nm) [7] in the literature.

Because of their size in the range of 1–100 nm, nanotherapeutics have many advantages, e.g., large surface area, easy functionalization properties, low toxicity, escalated bioavailability, etc. [8,9,10]. With regard to their sorts used in therapeutic systems, nanomaterials can be divided into different types, e.g., lipid-based NMs, inorganic material-based NMs, synthetic polymer-based NMs, and peptide-based NMs [11,12,13,14]. Peptide-based NMs are frequently employed in applications for targeted drug and gene delivery, primarily due to their tunable surface properties. Surface charge is recognized as a critical parameter in delivery applications. The layer-by-layer (LbL) polyelectrolyte assembly method is a robust and modular technique used to manipulate the surface charge of NMs. LbL assembly relies on the electrostatic interactions for sequential deposition of alternately charged polyelectrolytes onto the surface. The cationization of the surface prolongs the plasma lifetime of NMs by facilitating endosomal escape, enhancing cellular uptake, and enabling more efficient gene and drug delivery [15,16,17]. Charged therapeutics, including nucleic acids and proteins, can be readily incorporated within the electrostatic layers for effective combination therapy [18]. Surface cationization is often achieved through polycations rich in amine groups (e.g., polyethylenimine (PEI) and polylysine (PLL)). Amine-rich polycations can lead to unwanted cytotoxicity, immunogenicity, and nonspecific tissue accumulation [19,20]. On the other hand, surface modification with high-molecular-weight polyelectrolyte chains leads to a considerable enlargement of NMs, creating a disadvantage [2]. As a nontoxic alternative solution to these problems, we conducted modifications on peptide NMs using divalent cations like Ca^2+^ ions to confer them with amine-free cationic properties. These divalent cations serve as double-sided directional adhesives between peptide NMs and the modification layer or the therapeutics to be transported [21,22,23].

Drug or gene delivery applications often require the release of NMs into the bloodstream, which is populated by blood cells and a plethora of small peptides, proteins, sugars, lipids, and complexes of all these molecules. When nanomaterials come into contact with biological fluids like blood, they dynamically adsorb biomolecules, such as proteins, lipids, carbohydrates, etc., onto their surfaces to minimize their high surface free energy. The adsorption of molecules onto NMs is described as “biocorona” formation [24,25]. This biocorona is a dynamic and complex interface interacting with cells and determines nanomaterials’ fate in biological environments. The biocorona layer masks the pristine surface characteristics of the original nanomaterials and modulates biological processes such as stability, biocompatibility, circulation lifetime, biodistribution, cellular uptake, and toxicity [26,27,28]. The phenomenon of conferring a “biological identity” to NMs through natural protein coronas can be employed as a strategy for the surface functionalization of nanomaterials for drug delivery applications [29]. Albumin, which is the most abundant blood protein (60% of the total protein content of plasma), is utilized as a protective coating to prevent plasma protein adsorption, reduce the cellular uptake of NPs by macrophages, and extend the circulation time of NMs [30,31]. Antibody pre-coating of NPs has also been investigated. Tonigold et al. demonstrated that the pre-adsorption of antibodies could be a promising approach to targeted NM-mediated delivery [32].

In this study, we initially investigated the effects of synthesis parameters on the size of diphenylalanineamide dipeptide-based nanomaterials (FFANMs). Optimal NM synthesis parameters were determined for possible uses in the field of drug and gene delivery. FFANMs of different sizes were synthesized and characterized for their morphological and structural properties using a scanning electron microscope (SEM), a transmission electron microscope (TEM), and liquid chromatography–mass spectrometry (LC-MS/MS). Subsequently, surface charge properties were modified using cationic ions, e.g., Ca^2+^, and the results were examined through dynamic light scattering (DLS) analyses. The interactions of cationized FFANMs with certain biomolecules were evaluated using DLS analyses. Corona formation and biocompatible surface functionalization were examined by the modification of FFANMs with albumin and IgG proteins and DNA molecules. On the other hand, the interaction of FFANMs with DNA molecules was also evaluated for possible gene delivery uses in the future. The cytotoxic effect and biocompatibility of plane and cationized FFANMs on HeLa and HT29 cell lines were determined through cell culture tests.

## 2. Experimental Parts

### 2.1. Materials

Diphenylalanine amide (FFA) hydrochloride salt was purchased from Bachem (Bubendorf, Switzerland). Hexafluoro-2-propanol (HFIP) was acquired from CovaChem (Loves Park, IL, USA). All remaining chemicals were obtained from Sigma-Aldrich Chemical Co. (St. Louis, MO, USA). The water used during the synthesis of nanomaterials and the washing process was purified using a Barnstead (Dubuque, IA, USA) ROpureLP^®^ reverse osmosis unit.

### 2.2. Methods

#### 2.2.1. Synthesis of FFA-Based Nanomaterials

Synthesis of FFANMs was carried out in two stages. First, cross-linking was achieved through the Schiff base reaction of FFA dipeptides with glutaraldehyde (GA), resulting in the formation of FFA-[GA]_n_-FFA dimers. These dimers served as the building blocks for the nanomaterials.

In the second step, these dimers self-assembled into nanomaterials in an aqueous environment through hydrophobic interactions and hydrogen bonds. FFA dipeptides were first treated with the cross-linker for different durations (e.g., 15 min, 30 min, 1 h, 4 h, 12 h, 24 h, 48 h, and 72 h), and then the resulting dimers were subjected to self-assembly in the aqueous environment. This allowed for the investigation of the impact of both covalent cross-linking and self-assembly durations on the size of the nanomaterials.

For this purpose, the FFA dipeptide was dissolved in hexafluoro-2-propanol (HFIP) at a concentration of 100 mg/mL. This resulted in a 3 mg FFA/30 μL HFIP stock solution. To this prepared stock solution, 0.6 mL of a 25% GA solution was added, and the time of GA addition was recorded as the cross-linking beginning stage. After the predetermined cross-linking time, the cross-linking process was terminated by adding 2.4 mL of pure water to the vials, and the self-assembly process was initiated. The solution was left in the dark overnight for this process to complete. After the designated time, the resulting NMs were subjected to washing with high-speed centrifugation at 20.000 rpm in pure water, removing any unreacted glutaraldehyde and HFIP from the environment. The resulting NMs were dispersed in 3 mL of pure water and stored at +4 ℃ for characterization studies. At the end of these processes, the optimal cross-linking time for achieving the desired FFANM size (≤100 nm) was determined to be 48 h. Characterization studies were then continued with the FFANMs synthesized under the determined optimal conditions.

#### 2.2.2. Decoration of FFA-Based Nanomaterials with Ca^2+^ Ions

FFANMs were treated with Ca^2+^ ions, which are biocompatible and abundant in living organisms, in order to adjust the surface charge balances before cell interaction. For this purpose, FFANMs were treated with Ca(NO_3_)_2_ solution at different concentrations, such as 500, 1000, and 2000 ppm, prepared in pure water, and their surface charges were evaluated in terms of zeta potential (mV).

#### 2.2.3. Corona Formation Studies of FFA-Based Nanomaterials

Corona formation studies have been conducted to examine the behavior of FFANMs towards biological molecules (e.g., human serum albumin (HSA), deoxyribonucleic acid (DNA), and immunoglobulin G (IgG)). For this purpose, FFA and FFA-Ca^2+^ NMs of 0.7 mg/mL concentrations were mixed with HSA, IgG, and DNA molecules in different Eppendorf’s with 0.01 M MOPS buffer (3-(*N*-morpholino) propanesulfonic acid) at pH 7.4. The concentrations interacting with NMs in corona formation experiments were performed as 7 mg/mL for HIgG, 0.035 mg/mL for DNA, and 35 mg/mL for HSA, and these concentrations were preferred because they are the average values of biological molecules in human blood. NMs were left to interact with biomolecules at 20 rpm on a rotator for 2 h. Then, they were centrifuged at 20.000 rpm for 5 minutes to ensure that NMs precipitated. The concentrations of biomolecules before and after interaction with NMs were monitored spectrophotometrically at 280 nm for proteins and at 260 nm for DNA.

#### 2.2.4. Characterization of FFA-Based Nanomaterials

The Beckman Coulter Allegra 64R model refrigerated centrifuge (Brea, CA, USA) was used for size adjustment and washing processes of nanomaterials. In order to prevent the aggregation of nanomaterials, a Bandelin Sonorex sonicator (Bandelin electronic GmbH & Co., Berlin, Germany) device was used. Corona formation studies of NMs were monitored with a UV–Vis spectrophotometer (Shimadzu, UV mini 1240, Kyoto, Japan). Transmission electron microscope (TEM) (Hitachi HT7700, Tokyo, Japan) and scanning electron microscope (SEM) instruments (EVO LS 10 ZEISS 5600 SEM, Tokyo, Japan) were used for shape and size analyses of NMs. For SEM analyses, before taking SEM photos, NMs were coated with gold–palladium (40:60). Liquid chromatography–mass spectrometry (LC-MS/MS) (TSQ Quantum Access Max, ThermoScientific, Waltham, MA, USA) was used to obtain information about the structure and molecular weight of the synthesized FFANMs. Dynamic light scattering (DLS) and zeta potential (ZP) measurements were performed in a pure water environment (viscosity: 0.8872 cP, refractive index (RI): 1.33, dielectric constant: 78.5) at 25 °C with a backscatter angle of 173° by using a Zetasizer Nano ZS instrument (Malvern Instruments, Malvern, UK).

#### 2.2.5. Cytotoxicity Studies of FFA-Based Nanomaterials

In order to examine the cytotoxic effect of FFA-based NMs, they were interacted with HeLa (human cervical adenocarcinoma) and HT29 (human colon adenocarcinoma) cells. The cells were examined by the MTT method. MTT analysis is used to measure cellular metabolic activity as an indicator of cell viability, proliferation, and cytotoxicity. This colorimetric assay is based on the reduction of a yellow tetrazolium salt (3-(4,5-dimethylthiazol-2-yl)-2,5-diphenyltetrazolium bromide, MTT) by metabolically active cells to purple formazan crystals [33]. Living cells contain NAD(P)H-dependent oxidoreductase enzymes that reduce MTT to formazan [34]. Insoluble formazan crystals (formed on the plate) are dissolved using a solubilizing solution, and the resulting colored solution is quantified by measuring the absorbance at 540–720 nm using a spectrophotometer. The darker the solution, the greater the number of living and metabolically active cells. An MTT solution was prepared at a concentration of 0.5 mg/mL (25 mg/50 mL).

In the experiments, to determine the cytotoxic effect of FFANMs on HeLa and HT29 cell lines, the cells were placed into microplates at a concentration of 1 × 10^4^ cells/well (96 flat-bottomed wells at tissue culture grade). First, 100 µL of each prepared cell suspension was added to each well in modified DMEM (Dulbecco’s Modified Eagle Medium) culture medium, and after keeping them in the incubator for 24 and 72 h, NMs prepared at various concentrations of 18.0–36.0 µg/mL were added on the cell media.

## 3. Results and Discussion

### 3.1. Characterization of FFA-Based Nanomaterials

**Figure 1:**
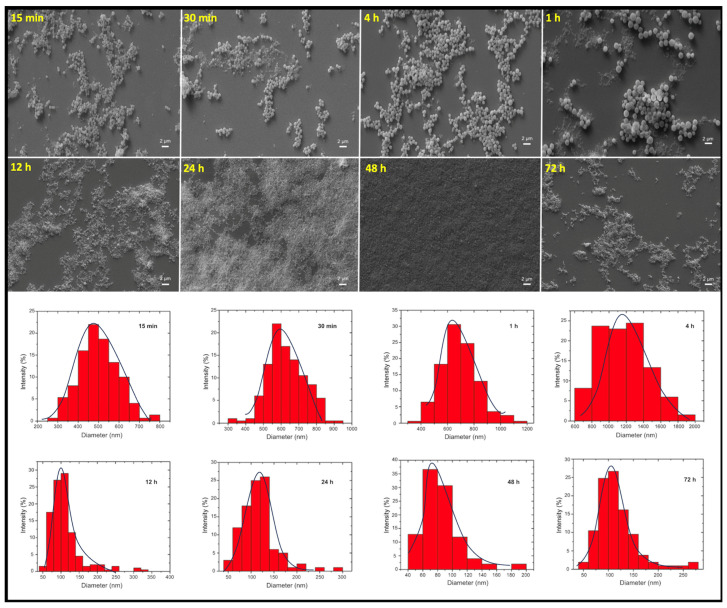
SEM images of FFANMs exposed to the cross-linker for different periods of time and the size–percentage distribution graphs.

The SEM images of FFANMs synthesized by exposure to the cross-linker for different periods of time and the size–percentage distribution graphs with Gaussian distribution obtained from these images with the Image J program (Version 1.54m) are given in Figure 1, and the average numerical values of the particle sizes are given in Table 1.

Table 1, where the results of the numerical values obtained using the SEM images in Figure 1 are given, shows that the average size of the FFANMs obtained as a result of the 48 h GA treatment is approximately 85 nm. It is seen that the average size of the FFANMs obtained as a result of the 12 and 24 h GA treatments is also approximately 100 nm, but when evaluated in terms of the average size distribution, it can be seen that the NMs obtained after the 48 h treatment is closer to monosize.

After the optimum time and conditions for synthesis were provided, the size–percentage graph and size distribution table obtained with the ImageJ program from the detailed SEM image of 48 h FFANMs are given in Figure S1 (Supplementary Materials). Figure S1A shows the SEM image of FFANMs with the evaluated size. The graphic and table of the size distribution analysis performed on the SEM micrograph with ImageJ are given in Figure S1B,C, respectively. Accordingly, the average diameter of NMs is 87.9 ± 20.4 nm (mean ± st deviation, n = 1000), and it is seen that 74% of the synthesized FFANMs are 100 nm and below. Most of the remaining 26% was analyzed to be around 110 nm.

Based on the data obtained, the polydispersity index (PDI) of FFANMs was calculated using Equation (1) and found to be 0.054. According to these results, it can be said that FFANMs are monodisperse [35].

(1) $$PDI={\left(Standart\;deviation/mean\;particle\right)}^{2}$$

For the dynamic light scattering (DLS) analysis of FFANMs, the obtained results are given in Figure S2 (Supplementary Materials). As seen in Figure S2, while the size results obtained from the dynamic light scattering graphs matched the results obtained from SEM (Figure S2A), the zeta potential of FFANMs was measured as −20.1 mV (Figure S2B).

**Figure 2:**
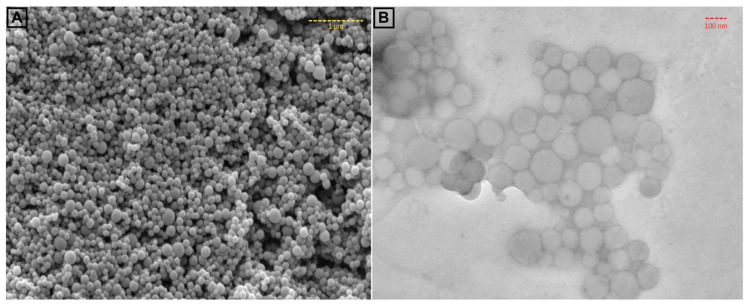
SEM (**A**) and TEM (**B**) images of FFA NMs.

In order to confirm the size of FFANMs, TEM images of NMs were also taken and are given in Figure 2. Here, the SEM image of NMs (Figure 2A) is also given along with TEM result (Figure 2B). In Figure 2, it is observed that the TEM analysis also supported the SEM results with regard to the size of FFANMs.

**Figure 3:**
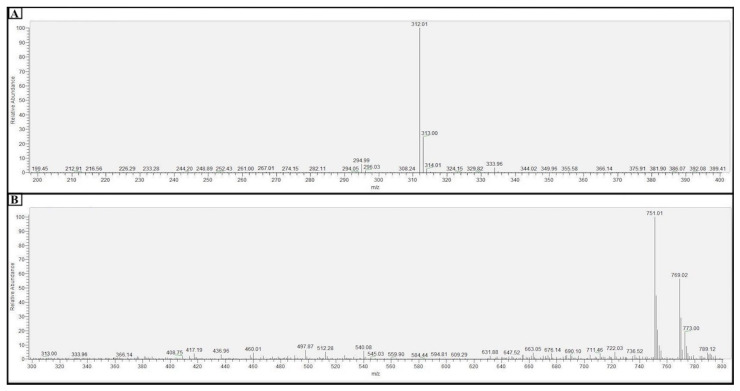
Result of the mass spectrometry (MS) analysis of FFA monomers (**A**) and FFANMs (**B**).

For the MS analysis, 1 mL samples of the FFA monomers and FFANMs were taken, and methanol (5 mL) was added to disperse them. These solutions were then passed through 0.45 µm filters and made ready for analysis. The mass spectroscopy analyses of FFA monomers and FFANMs are given in Figure 3.

When the findings obtained from LC-MSMS analysis for FFANMs are examined (Figure 3B), it is seen that FFA-[GA]_2_-FFA dimers (at 751.01 *m*/*z*) are formed at the end of the reaction of FFA monomers with the cross-linker GA for 48 h, and the uncross-linked FFAs (at 312 *m*/*z*) are not seen in the nanomaterial’s formation. These results demonstrate that the FFA monomers form dimer structures through cross-linking with GA, and these dimers self-assemble into spherical nanomaterials.

### 3.2. Decoration of FFA-Based Nanomaterials with Ca^2+^ Ions

**Figure 4:**
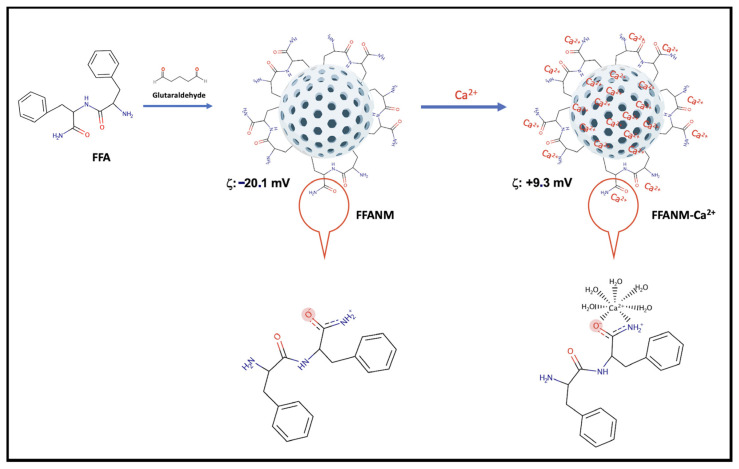
A model for the possible interaction between FFANMs and Ca^2+^ ions.

The surface charges of FFANMs were measured as −20.1 mV. To adjust the surface charges, FFANMs were treated with Ca(NO_3_)_2_ solution at different concentrations, such as 0.5, 1.0, and 2.0 mg/mL, prepared in pure water. The obtained zeta potentials (mV) are given in Table 2 and Figure S3 (Supplementary File). Additionally, a model for the possible interaction between FFANMs and Ca^2+^ ions is given in Figure 4.

As seen in Table 2 and Figure S3, while the surface charge of plain FFA-NMs was −20.1 mV, the surface charge potential shifted from a negative charge to a positive charge as Ca^2+^ ion concentrations increased. Corona tests and cell studies were continued with the NMs decorated at the concentration of 2.0 mg/mL because there was no significant change in zeta potential after this concentration.

Additionally, as seen in Figure 4, while the diphenylalanineamide (FFA) molecule normally appears uncharged, a resonance structure may form in the amide group depending on operating conditions, and the strong negative charge on oxygen can manifest itself in the molecule and, therefore, in NMs [2]. By adding Ca^2+^ ions to the medium, the negative charge on the surface is balanced, and a shift towards the positive charge occurs.

### 3.3. Corona Formation Studies of FFA-Based Nanomaterials

The structure formed on NMs after interaction with biological molecules is called corona formation, and this formation has some advantages for NMs. There are various studies focused on the positive effects of corona structures formed on NMs. NMs incubated with biological structures such as proteins can acquire a new biological identity. This new identity gained by NMs may enable them to receive a more positive immunogenic response, be targeted more efficiently, and be more stable against aggregation in working environments. For example, in a study with PEG NPs coated with Clusterin, the visibility of corona-formed NPs by macrophages was reduced [36]. In a different study, the aggregation rate of corona silver NPs formed with HSA was observed to decrease depending on the HSA concentration compared to bare NPs [37]. A more positive effect of the HSA corona-formed structures of antibody-bound NPs used for targeting was observed on human ovarian cancer cells [38].

**Figure 5:**
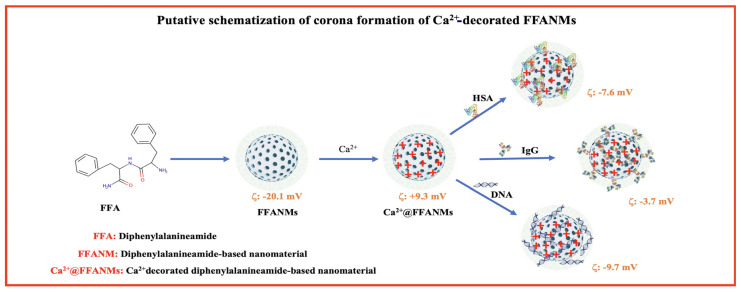
A putative representation of the corona formation of NMs with those molecules.

In this research, corona formation studies of FFANMs with human serum albumin (HSA), immunoglobulin G (IgG), and DNA were examined. A putative representation of the corona formation of NMs with those molecules is given in Figure 5, and the amounts of corona structures formed on NMs are given in Table 3. Additionally, the zeta potentials of the corona structures of NMs are given in Figure S4.

We can categorize the reactions of biological molecules to NMs in terms of corona formation into two groups. The first may be hydrodynamic, electrostatic, or steric forces, which are considered the main forces at the bio–nano interface, while the second is the size, shape, charge, surface ligands, modifications, and hydrophobicity and hydrophilicity of NMs [39]. Table 3 gives the amounts of HSA, IgG, and DNA adsorbing to plain FFANMs and Ca^2+^@FFANMs at the studied concentrations. As seen in Table 3, different biomolecules are adsorbed to NMs in varying amounts (here, g/g or mg /g ratios give the amount of biomolecules attached to 1 g of NMs). In corona formation, parameters such as the isoelectric points (p*I*) of biomolecules in the environment, their concentrations, the surface charge and size of the NM, and the size of the biomolecule forming the corona are of great importance [40,41]. IgG has a molecular weight of 150 kDa and a size of 10–15 nm [40], while HSA has a molecular weight of 66.5 kDa and a size of 8–13 nm [42]. In the studies we conducted, the large size of the protein showed its effectiveness in surface corona formation. The lower binding of IgG compared to HSA in corona formation may be attributed to the larger size of IgG. In addition, although the surface charge was negative, the adsorption of DNA molecules to positively charged NMs occurred at a low level (mg/g) due to the low concentration in the environment.

The zeta potential values of the corona-formed structures of Ca^2+^@FFANMs are given in Table S1 and Figure S4. As seen here, the zeta potential of NMs decorated with 2000 ppm-Ca^2+^ increased to +9.3, while the surface charges of NMs interacting with HSA, IgG, and DNA took the values of −7.6, −3.7, and −9.7 mV, respectively. These results show that NMs form corona in a physiological environment. Nucleotide chains are very strong polyelectrolyte chains. Molecules such as DNA and RNA play a strong role in changing the zeta potential, no matter how low the ambient concentrations are. The big change in zeta potential here is due to the strong polyelectrolyte structure of DNA. Since HSA and IgG are weak negative proteins and large proteins, we cannot expect them to change the surface charge as much as DNA, even though the amount interacting with NM is large.

**Figure 6:**
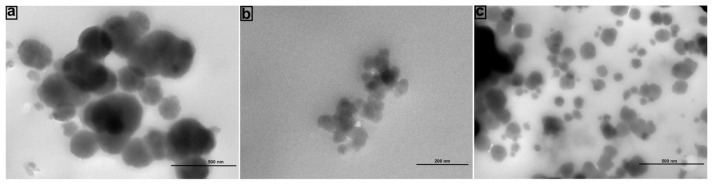
TEM analysis of corona structures formed after the interactions of Ca^2+^@FFANMs with HSA (**a**), IgG (**b**), and DNA (**c**).

TEM images of the corona structures formed by Ca^2+^@FFANMs interaction with HSA, IgG, and DNA are also given in Figure 6. TEM images of the corona structures of Ca^2+^@FFANMs formed with HSA, IgG, and DNA are marked as a, b, and c in Figure 6, respectively. In the TEM analyses examined, it is seen that corona formation does not cause a significant change in the dimensions of NMs. It is also seen that there is no deformation in the structure of NMs after corona formation. Keeping the structure stable provides a significant advantage for cell interactions [40]. When examining the TEM micrographs at the same concentration to assess the effect of corona formation on the aggregation of Ca^2+^@FFANMs, it can be stated that corona formation reduces the aggregation tendency of the NMs as compared to Figure 2B. Based on morphological examinations, it is observed that in Figure 2B, the NMs are aggregated, whereas aggregation is significantly reduced in Figure 6.

### 3.4. Cytotoxicity Studies of FFA-Based Nanomaterials

**Figure 7:**
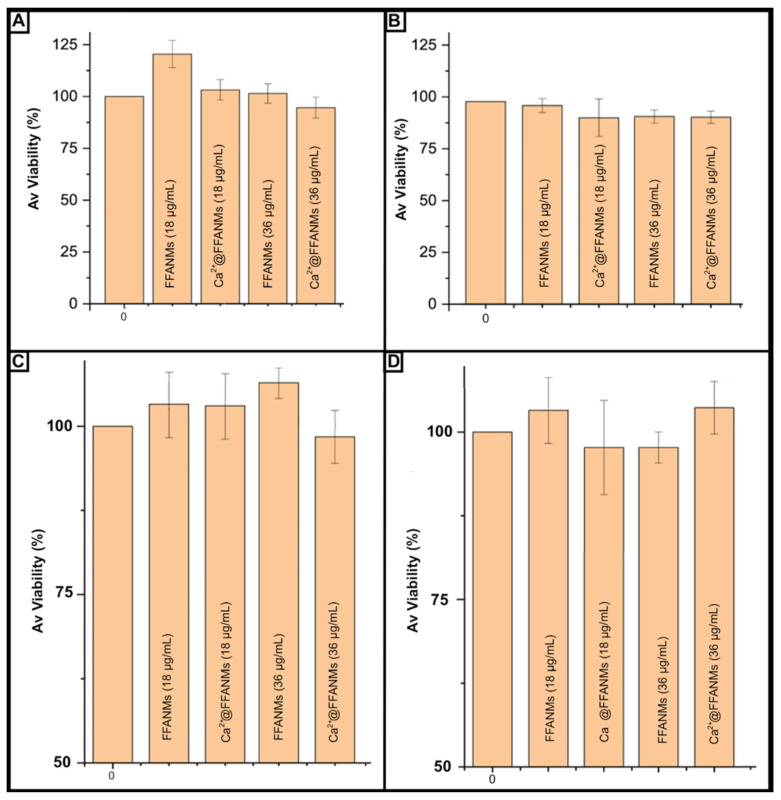
Viability percentages of cells after incubation with different concentrations of FFANM and Ca^2+^@FFANMs: 24 h (**A**) and 72 h (**B**) for HeLa cells; 24 h (**C**) and 72 h (**D**) for HT29 cells. Culture medium: DMEM; NM concentration range: 18–36 µg/mL; incubation temperature: 37 °C.

It is of great importance to perform biocompatibility tests of carrier sorbents prepared to be used in biological environments. To this end, in order to examine the cytotoxic effects of FFANM and Ca^2+^@FFANMs, NMs were allowed to interact with HeLa and HT29 cell lines for 24 and 72 h (Figure 7).

It was observed that the interacting cancer HeLa cell viability increased by 8% (for FFANM of 18 µg/mL) and decreased by 2% (Ca^2+^@FFANMs of 36 µg/mL) at the end of 24 h of incubation, depending on the NM derivatives and ratios in the medium (Figure 6a). After 72 h of incubation, a decrease of about 8% in HeLa cell viability was observed in the presence of FFANMs, especially including Ca^2+^ ions (Figure 6b). In the cytotoxicity tests resulting from the interactions of NMs with the HT29 cell line for 24 and 72 h, the decrease in HT29 cell viability was observed to be around 10% after 24 h of incubation (5% decrease for Ca^2+^@FFANMs of 36 µg/mL) (Figure 6c). After 72 h of incubation, a decrease of approximately 6% in cell viability was observed (Figure 7D). A subsequent increase in NM concentrations up to 220 μg/mL (72 h incubation) led to a decrease in the cytotoxicity of both cells from 100% to 88–90%. Thus, the nanomaterials examined showed themselves to be practically nontoxic materials toward human cell lines.

## 4. Conclusions

In this research, diphenylalanineamide dipeptide-based nanomaterials (FFANMs) were synthesized, decorated with Ca^2+^ ions to balance surface charge with regard to cationization, and characterized for possible use in gene delivery and drug release studies. The obtained data from SEM, TEM micrographs, and dynamic light scattering (DLS) results confirm that FFANMs were monosized and had an average diameter of 87.9 ± 20.4 nm. Corona formation tests of plain and Ca^2+^-decorated NMs were performed with two proteins (e.g., HSA and IgG) abundant in blood, and while the zeta potential of Ca^2+^@FFANMs was +9.3 mV, it was found to be −7.6 mV and −3.7 mV, for HSA and IgG corona-structured NMs, respectively. The interaction of Ca^2+^@FFANMs with DNA molecules was evaluated for possible gene delivery studies, and the shifting of zeta potentials from +9.3 mV to −9.7 mV confirmed the interactions. The cytotoxic effect and biocompatibility studies of plain and Ca^2+^-decorated NMs were practiced on HeLa and HT29 cell lines, and decreases of about 5% and 10% in viability at the end of 24 h and 72 h incubation times were determined. Finally, experiments performed in vitro indicated that the plain and Ca^2+^-decorated FFA-based NMs synthesized by our group are stable and biocompatible for possible use in cancer therapy and drug delivery studies. To the best of our knowledge, FFA-based nanomaterials have been synthesized for the first time in sizes around 80 nm for possible gene and drug delivery studies and have been examined with a comprehensive characterization. When compared to other protein- or amino acid-based NMs, we see that FFANMs are less complicated structures in terms of synthesis. From this perspective, we believe that scientists who will work on this subject will focus on these materials. The phenyl groups on the dipeptide allow the structures to self-assemble easily on top of each other. In addition, this study can be a guide for those who want to perform encapsulation studies with related molecules. The main idea of this study was to propose effective and nontoxic carriers for siRNA and drug delivery. In our next studies (unpublished data), we will show that these nanomaterials were able to effectively transport anticancer small interfering RNA into cancer cells and to induce the anticancer effects. The capability of present nanomaterials to deliver drugs (for example, hydrophobic anticancer drugs such as doxorubicin, methotrexate, and 5-fluorouracil) is a question of our next studies. We think that the results of this study assist the groups working in the relevant field.

## Figures and Tables

**Table 1:** Average value of the sizes of FFA-NMs exposed to the cross-linker for different periods of time.

GA Treatment Duration	FFANM Size (nm)
15 min	500.4 ± 44.2
30 min	631.5 ± 38.4
1 h	686.9 ± 28.9
4 h	117.9 ± 32.7
12 h	110.9 ± 22.8
24 h	116.5 ± 23.1
48 h	87.9 ± 20.4
72 h	117.1 ± 35.3

**Table 2:** Zeta potential values of FFANMs decorated with Ca^2+^ ions at different concentrations.

	FFANMs	FFANMs-Ca^2+^ 0.5 mg/mL Ca^2+^)	FFANMs-Ca^2+^ (1.0 mg/mL Ca^2+^)	FFANMs-Ca^2+^ (2.0 mg/mL Ca^2+^)
Zeta (mV)	−20.1	−10.5	+6.6	+9.3

**Table 3:** Interactions of FFANMs and Ca^2+^@FFANMs with HSA, IgG, and DNA.

C_HSA_ (mg/mL)	FFANMs/HSA (g/g)	Ca^2+^@FFANMs/HSA (g/g)
35	16.8	17.8
**C_IgG_ (mg/mL)**	**FFANMs/IgG (g/g)**	**Ca^2+^@FFANMs/IgG (g/g)**
7	12.8	12.6
**C_DNA_ (mg/mL)**	**FFANMs/DNA (mg/g)**	**Ca^2+^@FFANMs/DNA (mg/g)**
0.035	14.7	17.5

## Data Availability

The original contributions presented in this study are included in the article/Supplementary Materials. Further inquiries can be directed to the corresponding authors.

## Supplementary Materials

The following supporting information can be downloaded at: https://www.mdpi.com/article/10.3390/ma18010108/s1. Figure S1. Size evaluation via SEM image of FFANMs exposed to cross-linker for the determined optimum time (48 h); Figure S2. DLS analyses of FFANMs: size result (A), and zeta potential (B); Figure S3. The surface zeta potentials of FFANMs decorated with different Ca^2+^ ions concentration: Plane FFANMs (A); decoration with 0.5 mg/mL Ca^2+^ ions (B); decoration with 1.0 mg/mL Ca^2+^ ions (C); decoration with 2.0 mg/mL Ca^2+^ ions (D); Figure S4. Zeta potential values of the corona-formed structures of Ca^2+^@FFANMs; Table S1. Zeta potential values of corona-formed FFANMs-Ca^2+^.
