# Correction: Dikici et al. Synthesis, Characterization, and Investigation of Corona Formation of Dipeptide-Based Nanomaterials. *Materials* 2025, *18*, 108

**DOI:** 10.3390/ma19010074

**Published:** 2025-12-24

**Authors:** Emrah Dikici, Burcu Önal Acet, Betül Bozdoğan, Ömür Acet, Inessa Halets-Bui, Dzmitry Shcharbin, Mehmet Odabaşı

**Affiliations:** 1 Scientific and Technological Application and Research Centre, Aksaray University, Aksaray 68100, Turkey; emrah.dikici25@gmail.com; 2 Faculty of Arts and Science, Chemistry Department, Aksaray University, Aksaray 68100, Turkey; brconl33@gmail.com (B.Ö.A.); betul.bozdogan@gmail.com (B.B.); 3 Vocational School of Health Science, Pharmacy Services Program, Tarsus University, Tarsus 33100, Turkey; omuracetbio@gmail.com; 4 Institute of Biophysics and Cell Engineering of the National Academy of Sciences of Belarus, 220072 Minsk, Belarus; inessahalets@gmail.com

In the original publication [1], there was a mistake in Figure 6 as published. The corrected Figure 6 appears below. The authors state that the scientific conclusions are unaffected. This correction was approved by the Academic Editor. The original publication has also been updated.

## Figures and Tables

**Figure 6:**
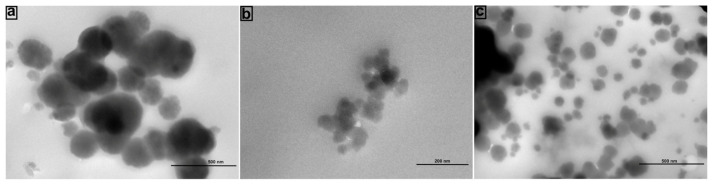
TEM analysis of corona structures formed after the interactions of Ca^2+^@FFANMs with HSA (**a**), IgG (**b**), and DNA (**c**).
