# Supplementary material for: Synthesis, Characterization, and Investigation of Corona Formation of Dipeptide-Based Nanomaterials
Source: Materials (Basel). 2024 Dec 30;18(1):108. doi: 10.3390/ma18010108 (PMC11721921; doi:10.3390/ma18010108)
Supplement: Supplementary file 1 [file materials-18-00108-s001.zip › materials-3376720-supplementary.pdf]

Supporting Information

Synthesis, Characterization, and Investigation of Corona Formation of Dipeptide-Based Nanomaterials

Emrah Dikici [1,2], Burcu Önal Acet [2], Betül Bozdoğan [2], Ömür Acet [3], Inessa Halets-Bui [4], Dzmitry Shcharbin [4,*] and Mehmet Odabaşı [2,*]

[1] *Scientific and Technological Application and Research Centre, Aksaray University, Aksaray, Turkey*
[2] *Faculty of Arts and Science, Chemistry Department, Aksaray University, Aksaray, Turkey*
[3] *Vocational School of Health Science, Pharmacy Services Program, Tarsus University, Tarsus, Turkey*
[4] *Institute of Biophysics and Cell Engineering of the National Academy of Sciences of Belarus, Minsk, Belarus*

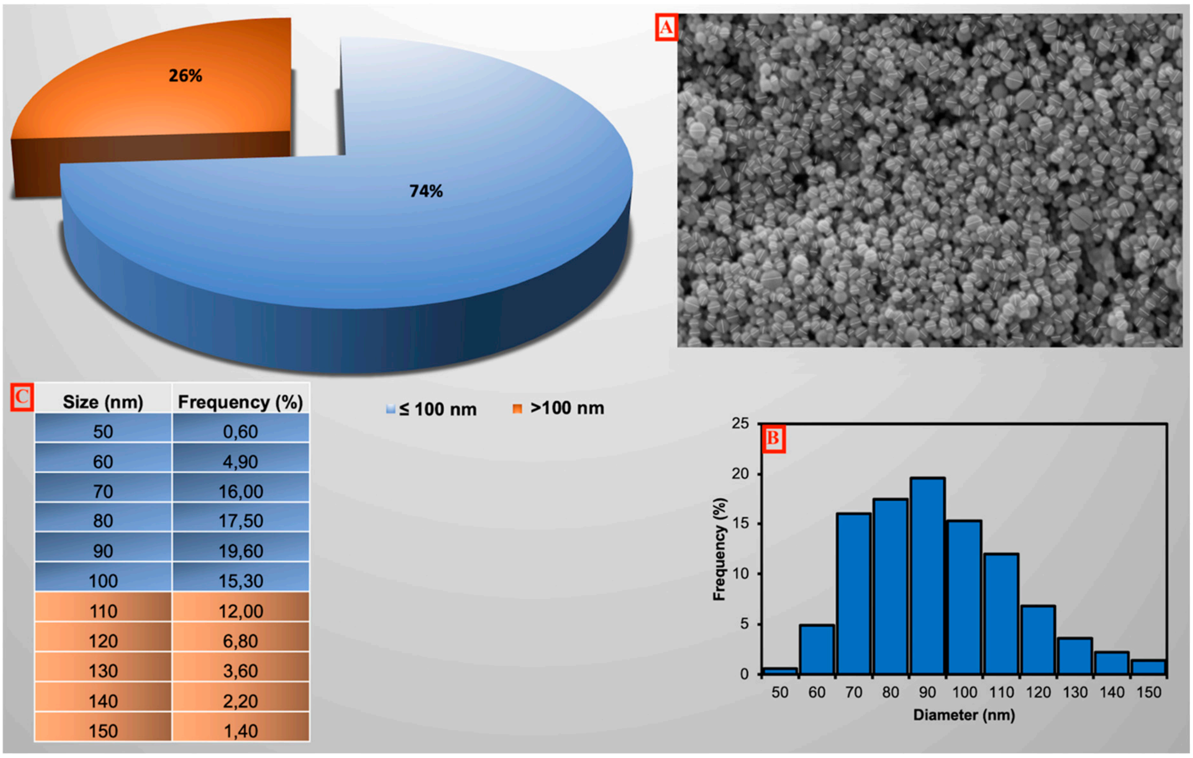

Figure S1. Size evaluation via SEM image of FFANMs exposed to cross-linker for the determined optimum time (48 h).

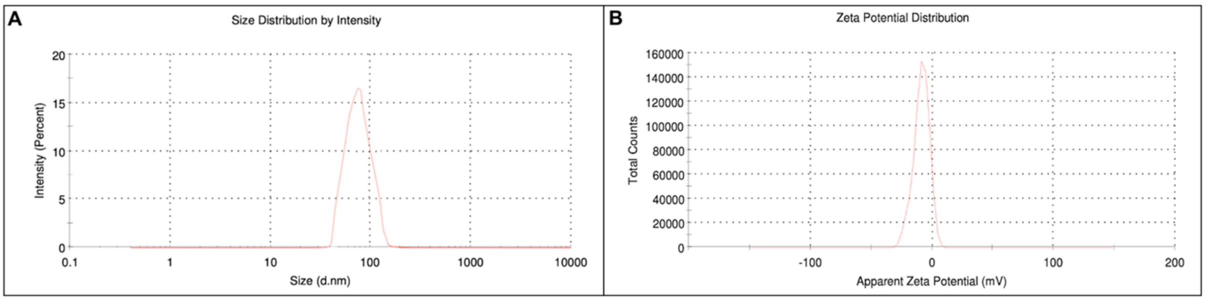

Figure S2. DLS analyses of FFANMs: size result (A), and zeta potential (B).

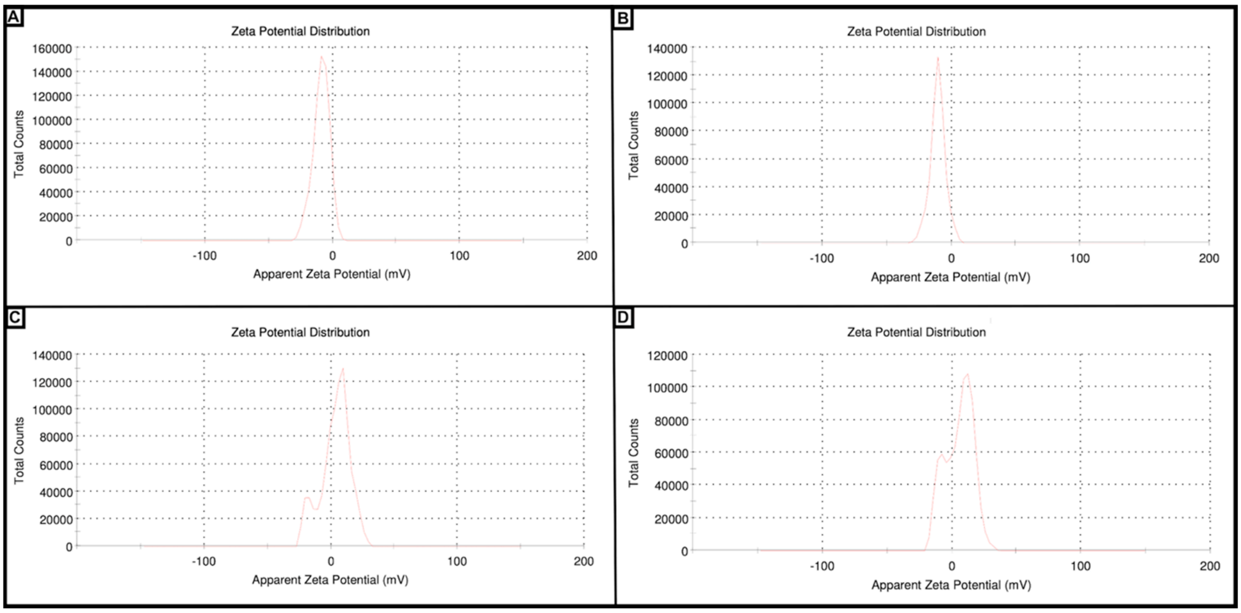

Figure S3. The surface zeta potentials of FFANMs decorated with different Ca^{2+} ions concentration: Plane FFANMs (A); decoration with 0.5 mg/mL Ca^{2+} ions (B); decoration with 1.0 mg/mL Ca^{2+} ions (C); decoration with 2.0 mg/mL Ca^{2+} ions (D).

Table S1. Zeta potential values of corona-formed FFANMs-Ca^{2+}.

	**FFANM-Ca^{2+}-(2000 ppm Ca^{2+})**	**FFA FFANMs-Ca^{2+}-HSA**	**FFA FFANMs-Ca^{2+}-IgG**	**FFA FFANMs-Ca^{2+}-DNA**
**Zeta (mV)**	+9,3	-7,6	-3,7	-9,7

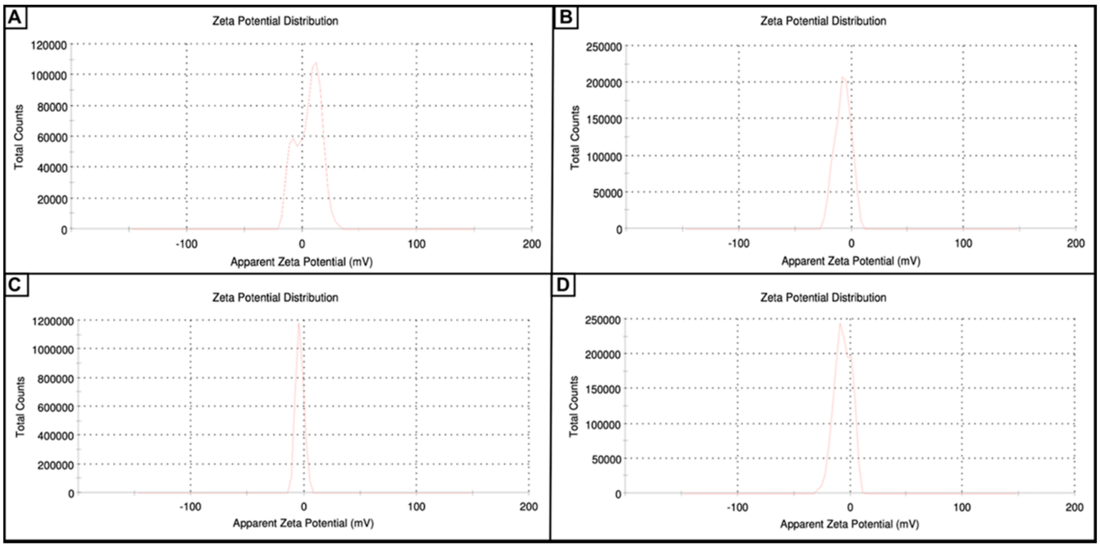

Figure S4. Zeta potential values of the corona-formed structures of Ca^{2+}@FFANMs.
